# A Educação como Determinante Social Associado ao Risco Cardiovascular

**DOI:** 10.36660/abc.20210444

**Published:** 2021-07-15

**Authors:** Dalton Bertolim Precoma

**Affiliations:** 1 Sociedade Hospitalar Angelina CaronDepartamento de Ensino e Pesquisa ClínicaCampina Grande do SulPRBrasilSociedade Hospitalar Angelina Caron - Departamento de Ensino e Pesquisa Clínica, Campina Grande do Sul, PR - Brasil; 2 Hospital Santa Cruz Rede D’Or CuritibaCuritibaPRBrasilHospital Santa Cruz Rede D’Or Curitiba - Cardiologia, Curitiba, PR - Brasil

**Keywords:** Educação, Mortalidade, Determinantes Sociais da Saúde, Doenças Cardiovasculares

A pandemia do SARS-Cov2 fez o mundo refletir sobre a importância do relacionamento entre os países na discussão dos problemas de saúde pública. As doenças chamadas crônico-degenerativas são universais e os países precisam agir de maneira conjunta para o melhor enfrentamento dos problemas. Esta promoção da saúde, ganhou destaque na primeira conferência internacional ocorrida em 1986 em Ottawa, visando a melhoria da saúde da população. Os chamados determinantes sociais (DS)^1^ incluíram a educação, habitação, alimentação, renda, ecossistema estável, recursos sustentáveis, justiça social, equidade e paz. Em 2005 a Organização Mundial da Saúde (OMS) elaborou uma Comissão para o delineamento dos determinantes sociais da saúde (DSS), para influenciar os governantes e a sociedade e chamar atenção ao enfrentamento dos fatores sociais que culminam com a desigualdade nos vulneráveis.^2^ E também definiu os DSS de forma bastante ampla como “as circunstâncias em que as pessoas nascem, crescem, vivem, trabalham e envelhecem, e os sistemas implantados para lidar com a doença”. Em um documento mais recente, a OMS ressalta a educação como fundamental fator associado a saúde da população.^3^

Em 2015, o “American Heart Association” publicou um documento estabelecendo o conceito dos DS para as doenças cardiovasculares. Este posicionamento se baseou nos fatores socioeconômicos (incluindo riqueza e renda), educação, emprego, raça, etnia, suporte social (incluindo as redes sociais), cultura, acesso aos cuidados médicos e ambientes residenciais. Estes fatores são muito inter-relacionados, sendo os fatores socioeconômicos os maiores determinantes (em torno de três quartos) e os demais, como os genéticos, biológicos e comportamentais, contribuindo com a menor parte.^4-6^

Preocupados com o tema dos DS relacionados com as doenças cardiovasculares (DCV) em nosso país, que possui vários contrastes sociais e de dimensões continentais, a Sociedade Brasileira de Cardiologia atualizou a Diretriz de Prevenção Cardiovascular, publicada em 2019 e inseriu um tópico específico para tratar este assunto. Direcionado aos gestores de saúde e a comunidade científica traz à tona a Educação e os aspectos associados a outros DS, como forma de redução das DCV.^7^

Apesar de aparentemente óbvio, o tema da educação isolada como DS e a associação com a DCV, é pouco explorado, e o estudo de Barreto et al.8 foi um dos primeiros que abordam a educação em relação ao infarto agudo do miocárdio num país em desenvolvimento. Neste artigo, foram incluídos 542 pacientes internados com infarto agudo do miocárdio com supradesnivelamento do segmento ST, num período aproximado de 10 anos. Foram considerados o maior nível de escolaridade atingido pelo paciente e divididos em quartis sendo o menor grau entre 0-3 anos de escolaridade e o maior grau acima de 10 anos. A média de acompanhamento foi de 21 meses e o grau médio de escolaridade 6,63 ± 4,94 anos. Na análise linear a mortalidade foi maior no grupo de menor escolaridade. Na análise univariada os fatores que influenciaram na mortalidade foram a idade, tabagismo, classificação de Killip e escolaridade. Quando avaliado pela análise multivariada a escolaridade e outras não foram significativas, prevalecendo somente a classificação de Killip.^8^

Apesar deste estudo demonstrar que a escolaridade não teve relação independente com a taxa de mortalidade pós infarto agudo do miocárdio (IAM), vários estudos citam a importância do nível de educação na evolução da doença coronariana.^9,10^ Associada a vários fatores de risco, a escolaridade que é associada a renda familiar, influencia na aderência ao tratamento, no acesso a medicações com maior eficácia e segurança, maior acesso a exames complementares adequados, além, de outros elementos que impactam na sobrevida do paciente.^7,9,10^

Do ponto de vista da educação, as Nações Unidas declaram que a educação é um direito humano. O artigo 26 da Declaração Universal dos Direitos Humanos cita que todos tem direito a educação e deve ser gratuita pelo menos na etapa elementar e fundamental. Que a educação será direcionada para o desenvolvimento da personalidade humana e para o fortalecimento do respeito aos direitos humanos e às liberdades fundamentais.^11^

No Brasil, alguns estudos apontam um maior número de mortalidade por DCV associada ao nível socioeconômico, como o de Piegas et al.^12^ ao analisarem os fatores de risco para o IAM no estudo AFIRMAR, realizado em 104 hospitais e em 51 cidades do Brasil, que encontraram relação com a educação e renda familiar como fatores para o aumento do infarto do miocárdio.^12^ Bassanesi et al.^13^ em estudo realizado em 73 distritos de Porto Alegre, apontaram que mais da metade da mortalidade por DCV em idade inferior a 65 anos pode ser atribuída a pobreza.^13^Soares et al.^14^ estudaram os indicadores socioeconômicos e da mortalidade cardiovascular nos estados do Rio de Janeiro, São Paulo e Rio Grande do Sul, e encontraram forte correlação na redução das DCV associadas ao aumento do produto interno bruto (PIB) e escolaridade.^14^

Estudos internacionais, abordaram a educação no contexto dos DS, como Janßen et al.,^15^ que realizaram uma revisão sistemática de 20 estudos sobre desigualdades sociais e prevenção em saúde na Alemanha. Demonstraram uma significativa associação entre morbidade e mortalidade e as desigualdades denominadas horizontais (idade, gênero, estado marital e nacionalidade) e verticais (ocupação, educação e renda).^15^Na Índia, Jeemon et al.^16^ confirmaram a associação do baixo nível socioeconômico com a presença de maior morbidade e mortalidade pelas doenças cardiovasculares e diabetes, observaram a ocorrência de 52% das mortes por DCV com a idade inferior a 70 anos. Os grupos de maior vulnerabilidade foram os de baixa escolaridade em comunidades mais urbanizadas.^16^

## Considerações finais

Atualmente é bem estabelecida a importância dos DSS e a doença cardiovascular. Apesar dos modernos recursos diagnósticos e de tratamento, a implementação das medidas preventivas pode ser muito afetada pela não observância dos múltiplos fatores que compõe os determinantes sociais.

É fundamental que os outros DSS sejam valorizados e estudados de maneira mais ampla e para que seja estabelecido a real dimensão do problema e soluções sejam propostas. Os países devem proporcionar a discussão mais ampla e colaborar na tomada de decisões de forma conjunta.
