# Supplementary material for: A Educação como Determinante Social Associado ao Risco Cardiovascular
Source: Arq Bras Cardiol. 2021 Jul 15;117(1):13–4. [Article in Portuguese] doi: 10.36660/abc.20210444 (PMC8294730; doi:10.36660/abc.20210444)
Supplement: Translation

# Translation: Education as a Social Determinant Associated with Cardiovascular Risk

**Authors:** Dalton Bertolim Precoma

**Affiliations:** 1 BrasilSociedade Hospitalar Angelina Caron - Departamento de Ensino e Pesquisa Clínica, Campina Grande do Sul, PR - Brasil; 2 BrasilHospital Santa Cruz Rede D’Or Curitiba - Cardiologia, Curitiba, PR - Brasil

**Keywords:** Educação, Mortalidade, Determinantes Sociais da Saúde, Doenças Cardiovasculares

The SARS-Cov2 pandemic has caused the world to reflect on the importance of the relationship between countries regarding the discussion of public health problems. Chronic-degenerative diseases are universal, and countries need to act together to better face the problems. This health promotion was highlighted at the first international conference held in 1986 in Ottawa, aimed at improving the population’s health. The so-called social determinants (SD)^1^ included education, housing, food, income, a stable ecosystem, sustainable resources, social justice, equity and peace. In 2005, the World Health Organization (WHO) created a Commission to outline the social determinants of health (SDH), to influence government and society, and draw attention to coping with social factors that culminate in inequality for vulnerable individuals.^2^ It also defined SDH quite broadly as “the circumstances in which people are born, grow, live, work and age, and the systems implemented to deal with the disease”. In a more recent document, the WHO emphasizes education as a fundamental factor associated with population health.^3^

In 2015, the American Heart Association published a document establishing the concept of SD for cardiovascular diseases. This position was based on socioeconomic factors (including wealth and income), education, employment, race, ethnicity, social support (including social networks), culture, access to medical care and residential environments. These factors are very interrelated, with socioeconomic factors being the main determinants (around three-quarters) and the others, such as genetic, biological and behavioral factors, contributing with the smallest part.^4-6^

Concerned with the topic of SD related to cardiovascular diseases (CVD) in our country, which has several social contrasts and continental dimensions, the Brazilian Society of Cardiology updated the Cardiovascular Prevention Guideline published in 2019 and included a specific topic to address this subject. Aimed at health managers and the scientific community, it brings up Education and the aspects associated with other SDs as a way to reduce CVD.^7^

Although apparently obvious, the topic of isolated education as an SD and the association with CVD is little explored, and the study by Barreto et al.^8^ was one of the first to address education in relation to acute myocardial infarction in a developing country. In this article, 542 patients hospitalized with ST-Segment Elevation Myocardial Infarction (STEMI) were included, during an approximate period of 10 years. The highest level of schooling attained by the patients was considered and divided into quartiles, with the lowest being between 0-3 years of schooling and the highest being >10 years of schooling. The mean time of follow-up was 21 months, and the mean level of schooling was 6.63 ± 4.94 years. In the linear analysis, mortality was higher in the group with fewer years of schooling. In the univariate analysis, the factors that influenced mortality were age, smoking, Killip classification and schooling. When assessed by the multivariate analysis, the level of schooling and others were not significant, prevailing only the Killip classification.^8^

Although this study demonstrates that schooling had no independent relationship with the mortality rate after acute myocardial infarction (AMI), several studies mention the importance of education for coronary heart disease evolution.^9,10^ Associated with several risk factors, the level of schooling, which is associated with family income influences adherence to treatment, access to medications with greater efficacy and safety, greater access to appropriate complementary exams, in addition to other elements that impact patient survival.^7,9,10^

From the point of view of education, the United Nations have declared that it constitutes a human right. Article 26 of the Universal Declaration of Human Rights states that everyone has the right to education, and that it should be free at least at the level of Elementary School and that the education shall be directed towards the development of the human personality and to strengthen the respect for human rights and fundamental freedoms.^11^

In Brazil, some studies indicate a higher number of CVD mortality associated with socioeconomic status, such as that by Piegas et al.,^12^ when analyzing the risk factors for AMI in the AFIRMAR study, carried out in 104 hospitals and in 51 cities in Brazil, which found a relationship with education and family income as factors for the increase in myocardial infarction.^12^ Bassanesi et al.,^13^ in a study carried out in 73 districts of Porto Alegre, found that more than half of CVD mortality cases under the age of 65 years could be attributed to poverty.^13^ Soares et al.^14^ studied the socioeconomic and cardiovascular mortality indicators in the states of Rio de Janeiro, São Paulo and Rio Grande do Sul, and found a strong correlation between CVD reduction and the increase in gross domestic product (GDP) and level of schooling.^14^

International studies have addressed education in the context of SD, such as Janßen et al.,^15^ who carried out a systematic review of 20 studies on social inequalities and health prevention in Germany. They demonstrated a significant association between morbidity and mortality and the so-called “horizontal” (age, gender, marital status and nationality) and “vertical” inequalities (occupation, education and income).^15^ In India, Jeemon et al.^16^ confirmed the association of low socioeconomic level with the presence of greater morbidity and mortality due to cardiovascular diseases and diabetes and observed the occurrence of 52% of deaths from CVD at the age < 70 years. The most vulnerable groups were those with low level of schooling in more urbanized communities.^16^

## Final considerations

Currently, the importance of SDH and cardiovascular disease is well established. Despite the modern diagnostic and treatment resources, the implementation of preventive measures can be greatly affected by the non-observance of the multiple factors that comprise the social determinants.

It is essential that the other SDH are taken into account and more broadly studied, aiming to establish the real dimension of the problem and then, solutions can be proposed. Countries must provide the broadest discussion and collaborate in joint decision-making.
